# Metabolic Pathways for Observed Impacts of Crop Load on Floral Induction in Apple

**DOI:** 10.3390/ijms23116019

**Published:** 2022-05-27

**Authors:** Priyanka Reddy, Tim Plozza, Vilnis Ezernieks, Dario Stefanelli, Alessio Scalisi, Ian Goodwin, Simone Rochfort

**Affiliations:** 1 Agriculture Victoria, AgriBio, Centre for AgriBioscience, Bundoora, VIC 3083, Australia; priyanka.reddy@agriculture.vic.gov.au (P.R.); tim.plozza@agriculture.vic.gov.au (T.P.); vilnis.ezernieks@agriculture.vic.gov.au (V.E.); 2 Manjimup Horticulture Research Centre, Department of Primary Industries and Regional Development, Manjimup, WA 6258, Australia; dario.stefanelli@dpird.wa.gov.au; 3 Tatura SmartFarm, Agriculture Victoria, Tatura, VIC 3616, Australia; alessio.scalisi@agriculture.vic.gov.au (A.S.); ian.goodwin@agriculture.vic.gov.au (I.G.); 4 Centre for Agricultural Innovation, University of Melbourne, Parkville, VIC 3010, Australia; 5 School of Applied Systems Biology, La Trobe University, Bundoora, VIC 3083, Australia

**Keywords:** chemical signalling, metabolomics, apple bud, biennial bearing, *Malus domestica* Borkh, plant hormones, return bloom

## Abstract

The triggers of biennial bearing are thought to coincide with embryonic development in apple and occurs within the first 70 days after full bloom (DAFB). Strong evidence suggests hormonal signals are perceived by vegetative apple spur buds to induce flowering. The hormonal response is typically referred to as the floral induction (FI) phase in bud meristem development. To determine the metabolic pathways activated in FI, young trees of the biennial bearing cultivar ‘Nicoter’ and the less susceptible cultivar ‘Rosy Glow’ were forced into an alternate cropping cycle over five years and an inverse relationship of crop load and return bloom was established. Buds were collected over a four-week duration within 70 DAFB from trees that had maintained a four-year biennial bearing cycle. Metabolomics profiling was undertaken to determine the differentially expressed pathways and key signalling molecules associated with biennial bearing. Marked metabolic differences were observed in trees with high and low crop load treatments. Significant effects were detected in members of the phenylpropanoid pathway comprising hydroxycinnamates, salicylates, salicylic acid biosynthetic pathway intermediates and flavanols. This study identifies plant hormones associated with FI in apples using functional metabolomics analysis.

## 1. Introduction

Apple is globally one of the most valued fruit crops and is of high economic importance to the horticultural industry [1]. Apple production, however, is significantly impacted by biennial bearing, a phenomenon that has been studied and reported since the early twentieth century [2]. The cause of alternate bearing is still largely unknown [3] and typically occurs in a tree or branch that does not yield a consistent crop load every year but alternates between a heavy and extremely light one. This common irregularity occurs in both deciduous and evergreen trees, and is reported in nuts, temperate fruits, tropical fruits and forest trees [2]. Fruit growers require a consistent crop load with good quality fruit that attracts a high market value. Several different horticultural practices are implemented to achieve a viable industry. These include crop management strategies such as application of nutrients, pruning and seasonal chemical or hand thinning. Yet, even with the best practices, trees can enter a biennial bearing cycle. A high crop load tree (“ON”) can result in poor quality fruit. Fruit size, color appearance, firmness, sugars and acidity can correlate negatively with high crop load levels [4]. These trees would be required to be adequately thinned to improve fruit quality for the current season but also yield a consistent crop load for the following season. Light crop load trees (“OFF”) are associated with bigger fruit sizes that can have storage disorders [5]. Both phases of the cycle cause a disruption in cropping levels resulting in serious economic losses for the apple industry and can cause significant financial strain on fruit growers.

Histological analysis has shown that the initial development of a flower bud coincides with a developing embryo or fruitlet [6]. The developing seed in young fruitlets is believed to repress flower induction in vegetative buds via phytohormones and removal of fruitlets at the 3–18 mm fruit size, through thinning practices have been shown to lead to a more consistent flower formation (return bloom) for the following season [3,7,8]. There have been investigations of other factors that influence flower formation in apple such as stress associated with temperature, photoperiod [9,10], water deficit as well as internal factors associated with carbon-nitrogen ratio, hormones and interaction with other organs (leaves, terminal shoot growth, and fruit) [3]. A recent investigation showed that return bloom can also be influenced by cultivar and rootstock, that differentially expressed sugars and hormones, suggesting that some cultivars and rootstocks are more susceptible to biennial bearing [8]. 

Flowering in apple involves four stages: floral induction (FI), flower bud initiation, differentiation, and floral bloom [3,6,11]. FI occurs when a chemical and/or environmental signal causes a vegetative bud to become floral. FI corresponds to a specific time when seeds from developing fruit are believed to emit signals to adjacent bud meristem; the time point at which this occurs can differ in apple cultivars [12]. Floral initiation is the period of histological changes that occur in the flower bud and the subsequent flower differentiation stage describes the visible morphological changes occurring in buds [6,11]. In trees with high crop load, flower bud development is hypothesised to be inhibited by chemical signals, leading to a biennial bearing cycle that is difficult to alter once the cycle is established [13,14]. 

Regulation of flowering was initially thought to be driven by nutritional competition between flower bud initiation and concurrent fruit formation [11]. An in vitro investigation showed that flower bud development in *Plumbago indica* was stimulated by disaccharides (sucrose, cellobiose or maltose) and certain plant hormone mixtures (cytokinins, adenine and low levels of auxin), and inhibited by amino acids (glutamine, asparagine), riboflavin and gibberellins [9,15]. More recently, genes [3], transcripts [6,16] and proteins [6] associated with biennial bearing were identified and studies indicated that a combination of increased carbohydrates and activation of FI genes, likely mediated by phytohormones, either stimulated or inhibited FI [6]. Hormone-related genes were likely candidates involved in biennial bearing compared to flowering genes. Moreover, transcriptomics studies showed that hormone responses were also differentially expressed. Plant hormone response to stress is linked to redox hub activity, such that ascorbic acid is either required for biosynthesis of plant hormones or low-levels promote accumulation [16]. A multi-omics investigation also indicated that thiamine, chlorogenic acid and an adenine derivative are involved in the metabolic pathway promoting early flower bud development in apple [6]. Increased levels of flavonoids such as kaempferol derivatives were also identified in low crop load trees.

To determine the metabolic pathways involved in FI, an untargeted metabolomics analysis of buds was utilised for this study. Buds were collected from young apple trees of the biennial bearing cultivar ‘Nicoter’ (marketed as Kanzi®) and the less susceptible to biennial bearing cultivar ‘Rosy Glow’ (marketed as Pink Lady®). The collection of buds was undertaken over eight weeks in the key time frame associated with floral induction as identified by Milyaev et al. [6], who found floral initiation to occur 75 and 97 days after flower bloom (DAFB) for ‘Fuji’ and ‘Gala’ with FI hypothesised to occur at least two weeks earlier. Polar extracts of the buds were analysed using high-resolution mass spectrometry and metabolite expression was compared across bud samples with various crop load treatments using multivariate and univariate analysis to identify key chemical determinants and associated disrupted metabolic pathways involved in inhibition or promotion of FI. 

The main objective of the present study was to contribute towards the largely unknown physiological mechanisms of biennial bearing in apple, thereby to better understand the underlying pathways and triggers of FI that might facilitate intervention opportunities for controlling apple crop load and thus ensuring stable apple production.

## 2. Results

**Figure 1:**
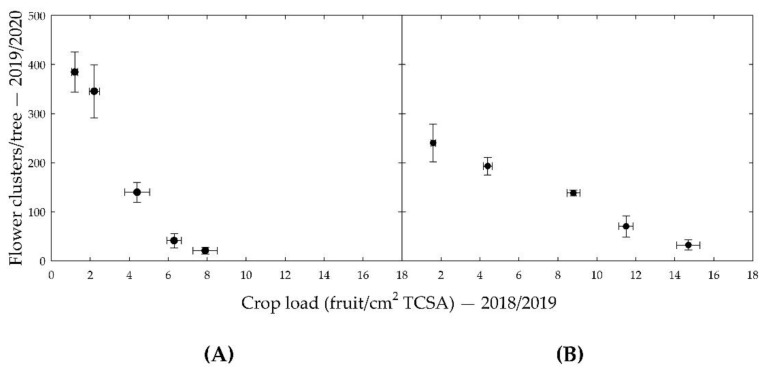
Flower clusters counted on trees in the 2019/2020 season against the crop loads (fruit/cm^2^ tree’s trunk cross sectional area (TCSA)) of those trees in the 2018/2019 season for: (**A**) ‘Nicoter’; (**B**) ‘Rosy Glow’. Each point is the average of three trees with standard error bars.

Strong inverse relationships between crop load in the 2018/2019 season and the number of flower clusters in the 2019/2020 season were found for variable crop load trees of both ‘Nicoter’ and ‘Rosy Glow’, as shown in Figure 1. For the constant crop load treatments, ‘Rosy Glow’ showed a similarly strong correlation to the variable treatment trees whereas ‘Nicoter’ showed a poor correlation. This was thought to be due to poor yield despite high crop load treatments (150% and 200%) and replacement trees due to disease at the beginning of the 2017/2018 and 2018/2019 seasons. For this reason, this study only focused on the variable crop load trees.

To investigate the physiological mechanisms of biennial bearing in apples, variable crop load treatments were applied and apple buds collected once a week over 8 weeks post-treatment were analysed using ultra-high performance liquid chromatography–high resolution mass spectrometry (UHPLC-HRMS).

Evaluation of the metabolomic data of ‘Nicoter’ and ‘Rosy Glow’ revealed a total of 908 compounds in positive ionisation mode and 668 compounds in negative ionization mode. Putative (Level 3) identification of 436 metabolites in the positive mode and 265 in the negative mode, provided valuable information on the complex composition of the apple bud, including plant hormones, lipids, amino acids, vitamins and phenols.

**Figure 2:**
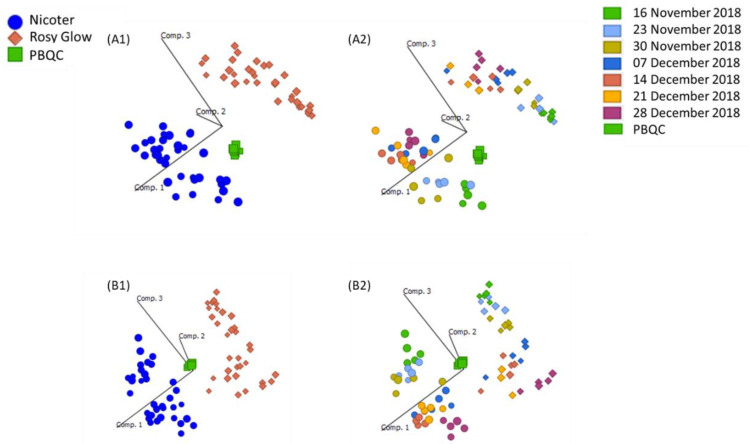
Principal component analysis (PCA) score plot showing ‘Nicoter’ (*n* = 35) and ‘Rosy Glow’ (*n* = 33) apple spur bud extracts evaluated on (**A**) ESI+ UHPLC-HRMS and (**B**) ESI- UHPLC-HRMS with a pooled biological quality control (PBQC) and categorised by (**A1**,**B1**) variety and (**A2**,**B2**) collection date.

Prior to statistical analysis of UHPLC-HRMS results, principal component analysis (PCA) plots were generated to assess the reproducibility of the pooled biological quality control (PBQC) samples for intensity drifts along the batch and no corrections were required. PCA plots of ‘Rosy Glow’ and ‘Nicoter’ (Figure 2) apple bud extracts revealed separation of the two cultivars in the positive (Figure 2A) and negative (Figure 2B) ionisation modes, indicating that the metabolomes of the individual cultivars are distinct. Although, crop load regulation is thought to be similar in cultivar [6], the PCA model indicates that the metabolome between the two cultivars may have differences and were thus explored individually. Furthermore, there may be differences in stress-induced pathways associated with apple trees treated with a variable crop load.

Initially, RV and NV datasets revealed no clear separation between treatment groups for RV (RV^HIGH^, RV^MID^, RV^LOW^) and NV (NV^HIGH^, NV^MID^, NV^LOW^) (Figure S1); however, some separation was observed between high and low treatment groups for each cultivar, particularly in the strong biennial cultivar ‘Nicoter’.

Thus, an orthogonal projection to latent structure discriminant analysis (OPLS-DA) model was applied to ‘Nicoter’ and ‘Rosy Glow’ high and low treatment groups for positive and negative whole datasets. The OPLS-DA score plot revealed discrimination between treatment groups in the positive and negative mode datasets.

**Figure 3:**
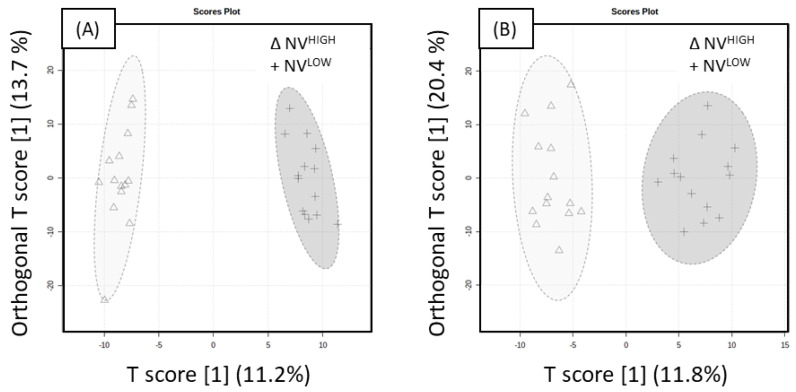
Orthogonal partial least squares discriminant analysis (OPLS-DA) of NV^LOW^ and NV^HIGH^ extracts acquired in UHPLC-HRMS. NV^LOW^ (+) and NV ^HIGH^ (Δ) OPLS-DA score plot for (**A**) ESI+ mode with an associated 95% confidence ellipses (Q^2^ = 0.601, R^2^Y = 0.724) and (**B**) ESI- mode with an associated 95% confidence ellipses (Q^2^ = 0.568, R^2^Y = 0.709). Both models were significant indicated by 100 different model permutations for (**A**) (*p* < 0.01, Q^2^ = 0.807 and R^2^Y = 0.983) and (**B**) (*p* < 0.01, Q^2^ = 0.816 and R^2^Y = 0.934).

The positive mode evaluation of model performance was well described with good predictive performance for NV^LOW^ and NV^HIGH^ (Q^2^ = 0.601, R^2^Y = 0.724), as shown in Figure 3A. The model was significant, as indicated by 100 different model permutations (Q^2^ = 0.807, *p* < 0.01 and R^2^Y = 0.983). The negative mode evaluation for NV^LOW^ and NV^HIGH^, shown in Figure 3B, revealed good predictive performance (Q^2^ = 0.568, R^2^Y = 0.709). The model was significant indicated by 100 different model permutations (*p* value < 0.01, Q^2^ = 0.816 and R^2^Y = 0.934). Compounds significantly responsible for the separation in OPLS-DA models were ascertained using the variable importance in projection (VIP) scores. A VIP > 1.5 was used as a cut-off for variable selection, resulting in a total of 72 significant metabolites in the positive mode and 69 metabolites in the negative mode. To further confirm the discrimination observed between the treatment groups, a linear model (y (metabolite response) ~ return bloom) was applied to the ‘Nicoter’ variable (NV^HIGH^, NV^MID^, NV^LOW^) for both positive and negative datasets. The return bloom data were treated as a covariate for each cultivar. The Benjamini–Hochberg (BH) correction was used to adjust the significance (*p* value) of each of the variables for the ‘Nicoter’ dataset, and the subsequent adjusted *p* value was referred to as a Q-value. The majority of significant metabolites in the apple spur buds showed higher levels in NV^HIGH^ treatments than NV^LOW^, as indicated in Table 1 and Table 2.

**Figure 4:**
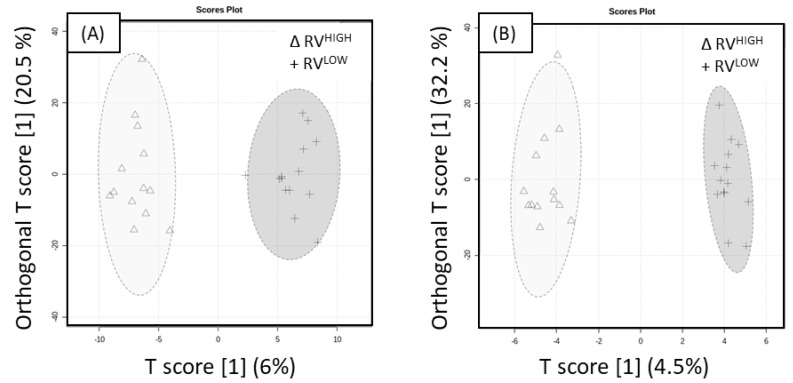
Orthogonal partial least squares discriminant analysis (OPLS-DA) of RV^LOW^ and RV^HIGH^ extracts acquired in UHPLC-HRMS. RV^LOW^ (+) and RV^HIGH^ (Δ) OPLS-DA score plot of (**A**) ESI+ mode with an associated 95% confidence ellipses (Q^2^ = 0.231, R^2^Y = 0.647) and (**B**) ESI- mode with an associated 95% confidence ellipses (Q^2^ = 0.071, R^2^Y = 0.593). Both models were significant indicated by 100 different model permutations for (**A**) (*p* < 0.01, Q^2^ = 0.757 and R^2^Y = 0.985) and (**B**) (*p* < 0.01, Q^2^ = 0.785 and R^2^Y = 0.983).

The OPLS-DA score plot of ‘Rosy Glow’ Variable UHPLC-HRMS ESI+ data with an associated 95% confidence ellipses demonstrates distinction between RV^HIGH^ and RV^LOW^ with lower predictive performance (Q^2^ = 0.231, R^2^Y = 0.647), as shown in Figure 4A. The model was significant indicated by 100 different model permutations (*p* value < 0.01, Q^2^ = 0.757, and R^2^Y = 0.985). The OPLS-DA score plot of ESI- with an associated 95% confidence ellipses demonstrating distinction between RV^HIGH^ and RV^LOW^, as shown in Figure 4B, revealed poor predictive performance (Q^2^ = 0.071, R^2^Y = 0.593). The model was significant indicated by 100 different model permutations (*p* value < 0.01, Q^2^ = 0.785 and R^2^Y = 0.983). A VIP > 1.5 cut-off was also applied to the ‘Rosy Glow’ variable and resulted in a total of 109 significant metabolites in the positive mode and 54 metabolites in the negative mode. A linear model (y (metabolite response) ~ return bloom) was applied to ‘Rosy Glow’ variable (RV^HIGH^, RV^MID^, RV^LOW^) to confirm the significance of the compounds. The return bloom data were treated as a continuous variable (covariate) for each cultivar. *p* values were only reported for the ‘Rosy Glow’ datasets as no significant Q values were obtained. The majority of significant metabolites in the apple spur buds showed higher levels in RV^HIGH^ treatments than RV^LOW^, as indicated in Table 3 and Table 4.

MS or MS^n^ fragmentation of parent ion confirmed the identification of differentially expressed metabolites for ‘Nicoter’ (Table 1 and Table 2) and ‘Rosy Glow’ (Table 3 and Table 4) variable treatments. Level 3 identification or above is required for putative identification of compounds in accordance with the Metabolomics Standards Initiative and Schrimpe-Rutledge et al. [17,18]. Most metabolites in Table 1, Table 2, Table 3 and Table 4 had level 2 identification—i.e., compounds that have matching fragmentation pattern with metabolite MS/MS libraries. Level 4 identification requires a unique molecular formula and level 3 requires the precursor m/z to match with a metabolite database. Thus, chlorogenic acid derivatives were assigned level 4 identification as some of the MS/MS ions matched the chlorogenic acid fragmentation data, although the precursor m/z did not match a database.

The majority of the differentially expressed metabolites were elevated in buds that were harvested from trees with high return bloom compared to low return bloom in both ‘Nicoter’ and ‘Rosy Glow’. In ‘Nicoter’, many of the phenylpropanoid pathway intermediates increased, comprising chlorogenic acid and its derivatives (I-VI) and precursor molecule quinic acid, coumarins (7-hydroxycoumarin, coumaranone), salicylates (3,4 dihydroxybenzaldehyde, salicylaldehyde) and a glycosidic derivative (4-acetyl-3-hydroxy-5-methylphenyl β-D-glucopyranoside), flavanols (afzelechin-7-apioside, naringenin, kaempferol) and flavanol glycosides (7-hydroxy-2-(4-hydroxyphenyl)-4-oxo-3,4-dihydro-2H-chromen-5-yl β-D-glucopyranoside; kaempferol-3-O-α-L-rhamnopyranoside). Few compounds including the flavanol (R)-shinanolone, glycosides of hydroxycinnamic acid derivatives 1-O-feruloylglucose and 3-O-acetyl-2-O-coumaroyl-hexopyranose and a methyl salicylate glycoside derivative showed significant decreases in ‘Nicoter’. Similarly, ‘Rosy Glow’ showed increased levels of phenylpropanoid pathway intermediates chlorogenic acid and its derivatives (VIII, IX and 3-caffeoyl-1,5-quinolactone) and precursor molecule quinic acid; coumarins and related derivatives (hydroxycoumarin; coumaric acid; dihydro-trans-o-coumaric acid 2-glucoside, coumaranone), glycosidic derivative of salicylate (2,5-dihydroxybenzoic acid 2-O-β-D-glucoside) and related phenolic glycoside (phlorisobutanophenone glycoside) and flavanol (kaempferol). Sugar alcohol D-(-)-mannitol also increased. The amino acids L-histidine and L-aspartate decreased in ‘Rosy Glow.’

**Figure 5:**
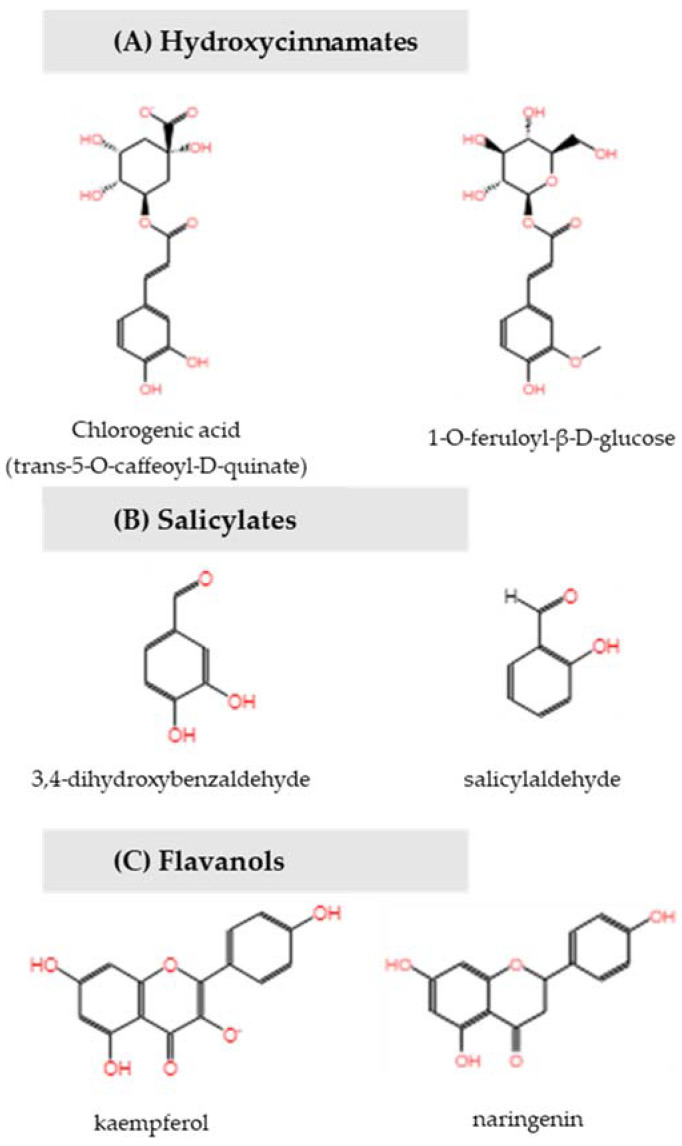
Compounds associated with the PAL (Phenylalanine Ammonia-Lyase) pathway to salicylic acid, including (**A**) hydroxycinnamates, (**B**) salicylates and (**C**) flavanols, show significant effects in ‘Nicoter’ and ‘Rosy Glow’ variable treatments associated with meristem bud formation.

Pathway enrichment analysis showed that compounds benzoate, 1-O-feruloyl-β-D-glucose, kaempferol and trans-5-O-caffeoyl-D-quinate (chlorogenic acid) belong to the phenylpropanoid derivative biosynthesis and this pathway is significantly disrupted (*p* = 0.005) (Figure 5). Increased levels of chlorogenic acid indicate significant disruption in the chlorogenic acid biosynthesis II pathway (*p* = 0.04) and the identification of unknown chlorogenic acid derivatives further increases its relevance.

Plant hormones including cytokinins, gibberellins and auxins are believed to participate in floral development in many plant species including apple [19,20]. To investigate the effect of crop load on selected phytohormones and their structural derivatives, relative abundances were measured for the cytokinin precursors, adenine and adenosine, auxins (2-oxindole-3-acetic acid (OxIAA), indole-3-acetic acid (IAA) indole-3-acetonitrile, methyl-indole-3-acetic acid (MeIAA) and precursors tryptophan (Trp) and tryptamine, gibberellic acid (GA), salicylic acid (SA), methyl jasmonate and abscisic acid (ABA). A line graph showing the effect of individual treatments on the compounds is shown for ‘Nicoter’ (Figure S2) and ‘Rosy Glow’ (Figure S3).

A linear model (y (metabolite response) ~ return bloom) was applied to ‘Rosy Glow’ (RV^HIGH^, RV^MID^, RV^LOW^) and ‘Nicoter’ (NV^HIGH^, NV^MID^, NV^LOW^) together with t-tests and fold change in high and low treatments. However, statistics tests revealed there was no significant differences for the selected compounds in ‘Nicoter’ (Table S1) and ‘Rosy Glow’ (Table S2).

Together these data show that increased biosynthesis of phenylpropanoid pathway intermediates, including hydroxycinnamates (chlorogenic acid, coumarates, ferulates), coumarins, salicylates and flavanols, increased in response to low crop loads.

## 3. Discussion

This study investigated the metabolic pathways of FI in apple using ESI LCMS metabolic profiling on apple buds collected within the critical 70 DAFB time period. In this investigation, levels of hydroxycinnamic acid derivatives, flavonoids and salicylates were significantly increased in buds collected from trees with low crop load (“OFF”) that expressed high return bloom in the following season. Although previous multi-omics investigations have identified differentially expressed genes associated with plant hormone signal transduction and significant metabolites such as flavanols and chlorogenic acid in “OFF” trees, there are no direct reports on the type of plant hormones regulating early flower bud development in apple [3,6,16]. 

To elucidate the metabolic pathways and key repressors and promotors of FI, young apple trees were forced into biennial bearing. Return bloom response for the 2019/2020 season was coupled to metabolomics profiling of apple spur buds collected in the previous 2018/2019 season using positive and negative ESI LCMS analysis to determine the physiological pathways disrupted during bud meristem development. Our results show that trees exhibiting an “OFF” season triggered metabolome changes that not only corroborated with previous studies but also identified novel candidates for FI belonging to the salicylate group of phytohormones and metabolites within the SA biosynthetic pathway. Biosynthetic production of SA occurs via isochorismate or phenylalanine in the model plant *Arabidopsis thaliana* [21]. Several metabolites were significantly disrupted by biennial bearing treatments imposed in this study, particularly those represented in Figure 5, providing evidence that the phenylalanine ammonia-lyase (PAL) pathway is likely triggered in the biosynthesis of SA and modification can render SA inactivate (e.g. glycosylation) or confer complementary properties such as activation of stress response via hydroxylation [21].

Guitton et al. described that the differentially expressed transcripts of apple trees in “ON” and “OFF” years showed that the redox and hormonal statuses are likely to contribute to FI in apple trees [16]. Disruption in the redox status of the plant is largely caused by reactive oxygen species (ROS) which are known to be toxic by-products of metabolic processes in normal conditions [22,23]. Under abiotic and biotic stress, there is a boost in production of ROS species which is believed to activate defense genes and the biosynthesis of SA for the activation of a defense response [22,24,25]. Redox homeostasis is maintained by enzymes and metabolites, and ascorbic acid is in the main line of defense for the detoxification of ROS species and leads to the biosynthesis of plant hormones, including SA [24]. Moreover, flavonoids are also considered as radical scavengers due to their antioxidative properties. In this study, increased levels of flavonoids kaempferol, naringenin and afzelechin were observed in “OFF” trees of the biennial bearing cultivar ’Nicoter’. Increased levels of kaempferol were also observed in ’Rosy Glow’. The sugar alcohol D-Mannitol can also function as a radical scavenger and was augmented exclusively in “OFF” trees of ’Rosy Glow‘. Sugar alcohols are produced in plants in response to abiotic and biotic stress and can provide plants with salinity tolerance, efficient growth and pathogen resistance [26].

The regulation of redox potential via antioxidants is involved in the signalling networks in both spatial and temporal dimensions of plant growth and development. ROS are strongly linked to the response to environmental factors, particularly stress and crosstalk with plant hormonal signalling pathways such as salicylates which are known to regulate floral transition [27,28]. Induction of early flowering ensures survival under stress [29]. 

The high-levels of hydroxycinnamic acid derivatives represented as chlorogenic acid, coumarate and ferulate derivatives in “OFF” trees indicated disruption in the phenylpropanoid biosynthesis pathway which was consistent with a previous study conducted by Milyaev et al. [6]. A gene homologue to caffeic acid metabolism, a likely metabolic precursor to the chlorogenic acid pathway, were also down-regulated in “ON” olive trees in a transcriptomic study [30]. Levels of salicylates were also observed to increase accordingly in the biennial cultivar ‘Nicoter’, resulting from decarboxylation of cinnamates. The elevated levels of chlorogenic acid and its derivatives suggest it is likely a key intermediate in FI, although it may not directly induce flowering [31]. Increased levels of salicylates are known to impact the flowering process in model plants such as duckweeds [27,28,31,32] and *Arabidopsis thaliana* [33,34], suggesting that a stress activation pathway is likely exerted in the bud, although benzoic acid was shown to elicit a more effective flower induction response than SA in duckweed [27]. Although SA (2-hydroxybenzoic acid) was not differentially expressed in this study, the closely related derivatives 3,4 dihydroxybenzaldehyde and salicylaldhehyde showed significantly increased levels in the biennial bearing cultivar ‘Nicoter’ and 2,5-dihydroxybenzoic acid 2-O-β-D-glucoside in ‘Rosy Glow’. 

The metabolite 4-hydroxycoumarin is also known to promote flowering in duckweed, and both cultivars ‘Nicoter’ and ‘Rosy Glow’ showed a significant increase in a hydroxycoumarin (unknown position of hydroxy group) [35]. Although there are no reports of flower-inducing activity of the closely related compound coumaranone, it may play an important role in FI, due to its significance and effect size reported in both cultivars. 

‘Rosy Glow’—the less susceptible cultivar to biennial bearing—showed low levels of amino acids histidine and aspartate in “OFF” trees, which are likely consumed to produce the dipeptide aspartyl-histidine which subsequently increased. In *Arabidopsis thaliana*, Histidine-to-Aspartate phosphorelays are involved in signal transduction induced by cytokinin and other environmental stimuli [36]. Cytokinins are a group of phytohormones that influence growth and the stimulation of cell division and are abundant in the root tip, shoot apex and all plant tissue. Although, cytokinins were not differentially expressed in this study, the significantly increased levels of aspartyl-histidine suggest that the His-Asp phosphorelays are likely a downstream effect of the perceived cytokinin signal [36]. Cytokinins are also responsible for activating the biosynthetic pathway for SA in plants [37] and when applied to apple buds post bloom at the time of fruit bud initiation, an increase in the amount of return bloom occurs [20,38]. The participation of the cytokinin class of phytohormones also corroborates with gene expression studies conducted by Milyaev et al. [6]. It is worth noting that, although no significant effects were observed in the auxin indole acetic acid (IAA) and gibberellic acid (GA), it is possible that phytohormone signalling cross-talk is occurring through mobile signals from the developing fruitlet seed or stem. Studies have indicated that fruit presence stimulates IAA transport in citrus and olive stem [39] and possibly induces GA biosynthesis. In apple, fruitlet seeds are rich in GA’s and application of GA is known to inhibit flowering [12,40]. 

Using ESI LCMS profiling, and MSMS fragmentation techniques, only 14% of the differentially expressed metabolites in ‘Nicoter’ and ‘Rosy Glow’ cultivars were identified. Albeit these were the most significant, further annotation of the “unknown” metabolites would be required to characterise the full extent of the metabolic pathways driving biennial bearing. 

In conclusion, to our knowledge, this is the first functional metabolomics analysis to identify plant hormones associated with FI in apple. This study showed that crop load treatments exerted significant effects on members of the phenylpropanoid pathway comprising hydroxycinnamates, salicylates, salicylic acid biosynthetic pathway intermediates and flavanols. Our findings provide evidence that the PAL salicylic acid biosynthetic pathway was activated in response to an “OFF” year during a biennial bearing cycle. Although no significant changes were exhibited in salicylic acid, its biosynthetic derivatives exhibited distinct increases in “OFF” trees of ‘Nicoter’ and ‘Rosy Glow’ apples. An additional mechanism of cytokinin involvement via histidine-aspartate phosphorelays’, which is also known to activate a defense response in trees, was observed in ‘Rosy Glow’. This study for the first time identifies the participation of the salicylate group of plant hormones in FI in apple.

## 4. Materials and Methods

The experiment was conducted in a commercial farm at Three Bridges, Yarra Valley, Victoria, Australia. Three-year-old trees of the cultivars ‘Nicoter’ (marketed as Kanzi^®^) and ‘Rosy Glow’ (marketed as Pink Lady^®^), trained on Open Tatura trellis, were used. Trees were managed according to the standard local practice and commercial operations. Five crop load treatments were first applied during the 2015–2016 growing season with six replicates, for a total of 30 trees per cultivar. Crop load treatments consisted of 1%, 50%, 100%, 150% and 200% of normal grower practice (6 fruit/cm2 for ‘Nicoter’, 8 fruit/cm^2^ for ‘Rosy Glow’), based on the tree’s trunk cross-sectional area (TCSA), which was measured 25 cm above the grafting union at the beginning of each growing season. In 2016–2017 and subsequent seasons, three replicates of each crop load treatment alternated between corresponding low and high treatments (e.g., 1% became 200%, 150% became 50%), thus forcing a biennial-type cropping behaviour on those trees (NV = ‘Nicoter’ variable crop load; RV = ‘Rosy Glow’ variable crop load).

During each season at full bloom (i.e., 80% open flowers), the number of flower clusters on each tree were counted manually to determine return bloom (RB). Flower clusters were then thinned by hand in an attempt to obtain the required number of fruit per tree. Only one flower per cluster was retained, except where insufficient clusters meant higher fruit numbers per cluster were needed to achieve the desired number of fruit per tree. Thinning was completed within 4 weeks of full bloom to minimize the chance of excess fruit affecting the following year’s return bloom.

The continuous return bloom variable was converted to a categorical dataset for ‘Nicoter’ and ‘Rosy Glow’ treatments denoted as: NV^HIGH^ (1–2 fruit/cm^2^ TCSA; 300–400 RB; *n* = 14) and RV^HIGH^ (1–4 fruit/cm^2^ TCSA; 190–240 RB; *n* = 13) = low crop load treatments, eliciting a high RB referred to as “OFF” trees. NV^LOW^ (6–8 fruit/cm^2^ TCSA; 20–41 RB; *n* = 14) and RV^LOW^ (11–15 fruit/cm^2^ TCSA; 32–70 RB; *n* = 14) = high crop load trees, eliciting a low return bloom, referred to as “ON” trees. Moderate treatments include NV^MID^ (4.43 fruit/cm^2^ TCSA; 139.7 RB; *n* = 14) and RV^MID^ (8.77 fruit/cm^2^ TCSA; 138 RB; *n* = 14).

### 4.1. Bud Selection and Preparation

In this study, buds were collected from the apple trees after thinning, in late spring and early summer of the 2018/2019 growing season. Weekly collection of one bud per tree began 4 weeks after full bloom and continued for 8 weeks. Buds were selected on spurs growing on at least two-year-old wood on lateral branches of the trees. Buds were prepared for molecular analysis by removing the scales from the bud using a scalpel then excising the growing tip containing the active meristem and immediately freezing it in dry ice in situ. Buds from each treatment were pooled into one 2 mL polypropylene mini centrifuge tube to ensure sufficient material for analysis. On return to the laboratory, these samples were stored at −80 °C until analysis.

### 4.2. Extraction of Metabolites from Buds

Samples collected in the first week were used for optimising the grinding and extraction methodology. Apple buds were lyophilised and subsequently one large (3.5–4.1 mm) and two small (2.8–3.2 mm) YTZP (yttria zirconia) beads were added to each tube. Samples were placed in 24-well cryo-blocks on a Geno/Grinder 2010 (SPEX Sample Prep, Metuchen, NJ, USA) and buds were ground at 1200 rpm for 1 min. The samples were extracted with 80% methanol/water (*v*/*v*), with extraction volumes adjusted proportionally to the weight of the lyophilised bud. Samples were centrifuged at 13,000 rpm for 2 min and 200 μL of the supernatant was transferred into a HPLC tube and stored at −20 °C until ready for LCMS analysis.

### 4.3. LCMS Methods for Untargeted and Targeted Analysis

For untargeted metabolite profiling, a Vanquish ultra-high performance liquid chromatography (UHPLC) system (Thermo Fisher Scientific, Bremen, Germany) with a binary pump, autosampler and temperature-controlled column compartment, coupled with a QExactive (QE) Plus mass spectrometer (Thermo, Bremen, Germany) with electrospray (ESI) probe operating in both positive and negative modes, was used. For MS data acquisition, positive and negative ion data were captured over a mass range of 80–1200 m/z, with a mass resolution set at 35,000. Samples were randomised, and blanks (80% methanol) injected every five samples. A pooled biological quality control (PBQC) was run every 10 samples. For MS^2^, data were acquired in full-scan MS/data-dependent MS^2^ (ddMS^2^) mode on positive and negative ionisation modes on selected samples. MS cycles were composed of 1 Full MS and up to 10 ddMS^2^. Ions within the inclusion list detected in the full MS survey scan (intensity threshold 1.6 × 10^5^) triggered a MS^2^ event at the peak apex with an isolation window of 0.4 m/z. A 5.0 s delay was required for the same ion to trigger a new MS^2^ event (dynamic exclusion). Full MS scans were acquired from m/z 100 to 1500 for the positive ionisation mode and 80 to 1200 for the negative ionisation mode with a resolution of 35,000 (full width at half maximum, FWHM, at m/z 200); automatic gain control (AGC) target was 3 × 10^6^; maximum injection time (IT) 200 ms. Scans (ddMS^2^) were acquired at a resolution of 17,500, the AGC target was 1 × 10^5^ and the maximum IT was 50 ms. Ions were fragmented with stepped collision energy (20, 40 and 60%).

Prior to data acquisition, the system was calibrated with Pierce LTQ Velos ESI Positive and Negative Ion Calibration Solution (Thermo Fisher Scientific). Mass spectrometry data were acquired using Thermo Xcalibur V. 2.1 (Thermo Fisher Scientific Inc., Waltham, MA, USA). Nitrogen was used as the sheath, auxiliary and sweep gases at flow rates of 28, 15 and 4 L/min, respectively. Spray voltage was set at 4000 V (positive and negative).

A Thermo Fisher Scientific Hypersil Gold 1.9 μm, 100 mm × 2.1 mm column with a gradient mobile phase consisting of 0.1% formic acid in H_2_O (A) and 0.1% formic acid in acetonitrile (B), at a flow rate of 0.3 mL/min was used. The gradient began at 2% B, increasing to 100% B over 11 min; followed by 4 min at 100% B before a 5 min equilibration with 2% B.

### 4.4. Data Processing and Statistical Analyses

The data files obtained following LCMS analyses were processed in the Refiner MS module of Genedata Expressionist^®^ 12.0 with the following parameters: (1) chromatogram chemical noise subtraction with removal of peaks with less than 4 scans, chromatogram smoothing using moving average estimator over 5 scans and 30% quantile over 151 scans for noise subtraction, (2) intensity thresholding using a clipping method and a threshold of 100,000, (3) selection of positive mode data only, (4) chromatogram RT alignment using a pairwise alignment-based tree and a maximum RT shift of 1 min, (5) chromatogram peak detection using a 5 scan summation window, a minimum peak size of 0.1 min, a maximum merge distance of 0.05 Da, a boundary merge strategy and a maximum gap/peak ratio of 70% with moving average smoothing over 10 scans for peak RT splitting, (6) a chromatogram isotope clustering using RT and m/z tolerance of 0.05 min and 0.05 Dalton, respectively, with a maximum charge of 2 and finally (7) an adduct detection using mainly M + H and allowable adducts (M + 2H, M + K, M + Na, M − H_2_O + H).

Statistical analyses were performed using the Analyst module of Genedata Expressionist^®^ 12.0. Principal component analyses (PCA) were performed to identify tissue and treatment differences. Overlay of the PBQC and samples allowed for the validation of the high-quality dataset by ensuring RT variation, mass error and sensitivity changes throughout. Identification of metabolites was performed by searching experimental MS^1^ data through the following databases: Plant Metabolic Network (PMN) https://plantcyc.org (accessed on 23 January 2022); Human Metabolome DataBase (HMDB) (http://hmdb.ca) (accessed on 10 June 2021); ChemSpider (http://chemspider.com) (accessed on 13 June 2021); and Lipid Maps^®^ (http://www.lipidmaps.org) (accessed on 20 June 2021). MS^2^ data were searched on MzCloud (https://www.mzcloud.org) (accessed on 30 June 2021). Identified significant compounds were inputted into a SmartTable in Plant Metabolic Network (PMN) https://plantcyc.org (accessed on 23 January 2022) for pathway enrichment analysis specific to *Malus domestica*.

A linear model (y (metabolite response) ~ return bloom) applied to the variable crop load treatments of the individual ‘Nicoter’ and ‘Rosy Glow’ cultivars, revealed significant metabolites. The Benjamini–Hochberg (BH) correction criteria were used to adjust the significance (*p* value) of each of the variables, and the subsequent adjusted *p* value is referred to as a Q-value.

Prior to multivariate analysis, the missing value imputation was applied and features with >10% missing values were removed and remaining missing values imputed by k-nearest neighbour (KNN) for each feature. Subsequently, a cube root transformation and autoscaling was applied to the data to achieve normality and homoscedasticity. An OPLSDA model was applied to each dataset using MetaboAnalyst 3.053 41 and Q^2^ value ≥ 0.4 indicates a model with good predictability.

For targeted analysis of plant hormones and structurally related compounds as described in Table 5, an extracted ion chromatogram (EIC) with a 5 ppm tolerance of [M + H] + was utilised to obtain the relative abundances from the MS spectra, in LCQUAN™ Quantitative Software (Thermo Fisher Scientific). All compounds were purchased from Sigma-Aldrich (St. Louis, MI, USA). Standards were prepared in 80% water/methanol.

Targeted analytes which resulted in *p* < 0.05 from a two-sided Student *t*-test and exhibited a log10 fold change of >0.5 in high and low treatments of ‘Nicoter’ and ‘Rosy Glow’ were regarded as significant.

## Figures and Tables

**Table 1:** Metabolites identified in UHPLC-HRMS ESI+ data that were significant (VIP > 1.5) in the OPLSDA model between NV^HIGH^ compared to NV^LOW^ apple spur buds with associated effect size. Benjamini–Hochberg adjusted *p* values (Q-values) indicate those metabolites that are significant in the linear model with the (y ~ return bloom) for NV^LOW^, NV^MID^ and NV^HIGH^.

Identity	Retention Time (min)	Mass (m/z) [M + H] +	Molecular Formula	Mass Error (ppm)	VIP Score	Effect Size	*	Q-Value (BH Adjusted *p* Value)	MS^2^ Ions	Metabolite Level
chlorogenic acid	3.68	355.1019	C_16_H_18_O_9_	−1.41	2.5	2.1	↑	1.4 × 10^−6^	163.0390, 145.0287, 135.0443, 117.0339	2
hydroxycoumarin	3.68	163.0389	C_9_H_6_O_3_	−0.43	2.5	2.0	↑	1.4 × 10^−6^	135.0442, 107.0495, 95.0498, 79.0391	2
coumaranone	3.68	135.0440	C_8_H_6_O_2_	−0.41	2.5	2.0	↑	1.5 × 10^−6^	117.0339, 107.0491, 89.0386	2
chlorogenic acid derivative I	3.67	728.1708	-	-	2.3	2.0	↑	8.9 × 10^−5^	374.0763, 551.1243, 747.1460, 163.0393,	4
chlorogenic acid derivative II	3.67	559.1093	-	-	2.3	1.9	↑	2.5 × 10^−5^	188.0710, 163.0394, 145.0287, 135.0444	4
chlorogenic acid derivative III	3.67	382.0613	-	-	2.3	1.7	↑	2.0 × 10^−5^	163.0393, 135.0446, 89.0392	4
chlorogenic acid derivative IV	3.71	645.1810	-	-	2.3	2.5	↑	2.5 × 10^−4^	291.0858, 163.0392, 139.0393	4
hydroxibenzoisochromanquinone derivative	4.15	230.0572	C_13_H_9_O_4_	−0.70	2.2	1.4	↑	9.3 × 10^−5^	147.0442, 119.0495, 91.0545	3
chlorogenic acid derivative V	3.68	551.1230	-	-	2.2	1.7	↑	1.3 × 10^−4^	163.0394, 145.0289, 135.0445, 117.0339, 89.0391	4
afzelechin 7-apioside	5.59	407.1336	C_20_H_22_O_9_	−0.14	2.1	1.3	↑	4.1 × 10^−3^	257.0814, 205.0495, 181.0498, 107.0497	2
chlorogenic acid derivative VI	3.68	217.5414	-		2.1	1.8		1.6 × 10^−4^	163.0392, 135.0442, 117.0337, 89.0388	4
3-O-acetyl-2-O-coumaroyl-hexopyranose	4.66	369.1180	C_17_H_20_O_9_	−0.02	2.1	1.6	↓	9.0 × 10^−3^	119.0494, 91.0547, 85.0289	2
(R)-shinanolone	4.66	193.0860	C_11_H_12_O_3_	0.40	2.0	1.5	↓	6.3 × 10^−3^	193.0861, 91.0546, 57.0340	2
7-hydroxy-2-(4-hydroxyphenyl)-4-oxo-3,4-dihydro-2H-chromen-5-yl β-D-glucopyranoside	5.43	435.1284	C_21_H_22_O_10_	−0.39	1.8	1.3	↑	1.7 × 10^−2^	271.0615, 151.0028, 119.0493, 107.0128	2
1-O-feruloylglucose	4.19	357.1176	C_16_H_20_O_9_	−1.14	1.7	1.6	↓	3.5 × 10^−2^	177.0546, 147.0443, 137.0598, 119.0495	2
3,4 dihydroxybenzaldehyde	4.04	139.0389	C_7_H_6_O_3_	−0.51	1.7	1.2	↑	3.4 × 10^−2^	111.0444, 93.0339, 83.0496	2
naringenin	5.43	273.0757	C_15_H_12_O_5_	−0.18	1.6	1.3	↑	3.6 × 10^−2^	231.0662, 153.0184, 147.0443, 119.0495	2
kaempferol-3-O-α-L-rhamnopyranoside	5.30	433.1125	C_21_H_20_O_10_	−0.98	1.6	1.3	↑	1.1 × 10^−2^	287.0556, 271.0603, 85.0289, 71.0497	2
kaempferol	5.24	287.0547	C_15_H_10_O_6_	−1.10	1.6	1.3	↑	2.9 × 10^−2^	213.0549, 153.0182, 121.0284	2
salicylaldehyde	4.04	123.0440	C_7_H_6_O_2_	−0.46	1.6	1.2	↑	4.8 × 10^−2^	95.0496, 77.0391, 67.0547, 53.0381	2

* ↑ = up-regulated in NV^HIGH^, ↓ = down-regulated in NV^HIGH.^

**Table 2:** Metabolites identified in UHPLC-HRMS ESI- data that were significant (VIP > 1.5) in the OPLSDA model between NV^HIGH^ compared to NV^LOW^ apple spur buds with associated effect size. Benjamini–Hochberg adjusted *p* values (Q-values) indicate those metabolites that are significant in the linear model with the (y ~ return bloom) for NV^LOW^, NV^MID^ and NV^HIGH^.

Identity	Retention Time (min)	Mass (m/z) [M − H] −	Molecular Formula	Mass Error (ppm)	VIP Score	Effect Size	*	Q-Value (BH Adjusted *p* Value)	MS2 Ions	Metabolite Level
quinic acid	3.68	191.0556	C_7_H_12_O_6_	−2.68	2.4	2.4	↑	1.4 × 10^−8^	191.0556, 173.0449, 127.0391, 82.0284	2
chlorogenic acid	3.68	353.0882	C_16_H_18_O_9_	1.12	2.3	2.4	↑	4.8 × 10^−8^	191.0556, 353.0886, 173.0450	2
4-acetyl-3-hydroxy-5-methylphenyl β-D-glucopyranoside	3.52	327.1092	C_15_H_20_O_8_	2.02	2.2	1.8	↑	8.3 × 10^−7^	165.0548, 147.0449, 163.0392, 121.0646, 119.0493	2
chlorogenic acid derivative VII	3.71	643.1680	-	-	2.2	2.8	↑	7.7 × 10^−6^	191.0567, 353.0878	4
quinic acid isomer	4.15	191.0556	C_7_H_12_O_6_	−2.68	2.2	2.1	↑	6.8 × 10^−6^	191.0556, 173.0449, 127.0391, 82.0284	2
Methyl salicylate glycoside derivative	4.33	461.1670	C_20_H_30_O_12_	1.19	2.1	1.8	↓	6.1 × 10^−4^	191.0555, 251.0773, 149.0455, 131.0340, 415.1611	2

* ↑ = up-regulated in NV^HIGH^, ↓ = down-regulated in NV^HIGH.^

**Table 3:** Metabolites identified in UHPLC-HRMS ESI+ data that were significant (VIP > 1.5) in the OPLSDA model between RV^HIGH^ compared to RV^LOW^ apple spur buds with associated effect size. *p* values indicate those metabolites that are significant in the linear model with the (y ~ returnbloom) for RV^LOW^, RV^MID^ and RV^HIGH^.

Identity	Retention Time (min)	Mass (m/z) [M + H] +	Molecular Formula	Mass Error (ppm)	VIP Score	Effect Size	*	*p* Value Linear Model	MS^2^ Ions	Metabolite Level
D-(-)-mannitol	1.23	183.0862	C_6_H_14_O_6_	−0.65	2.3	1.3	↑	5.2 × 10^−3^	181.0712, 101.0239, 89.02338, 71.0128	2
chlorogenic acid derivative VIII	3.68	775.1544	-		2.2	1.2	↑	4.5 × 10^−1^	709.0716, 532.0876, 421.0622, 163.0393	4
chlorogenic derivative IX	3.68	551.1230	-		1.9	1.6	↑	5.9 × 10^−2^	585.1142, 374.0770, 255.0183, 163.0394	4
kaempferol	5.24	287.0547	C_15_H_10_O_6_	−1.09	1.9	1.3	↑	2.9 × 10^−2^	213.0549, 153.0182, 121.0284	2
3-caffeoyl-1,5-quinolactone	4.17	337.0915	C_16_H_16_O_8_	−0.87	1.8	1.5	↑	3.2 × 10^−3^	163.0411, 145.0304, 135.0460, 117.0351	2
chlorogenic derivative X	3.67	728.1708	-		1.8	1.5	↑	2.8 × 10^−1^	551.1243, 374.0763, 163.0393	4
chlorogenic acid	3.68	355.1019	C_16_H_18_O_9_	−1.29	1.6	1.4	↑	1.3 × 10^−1^	163.0390, 145.0287, 135.0443, 117.0339	2
hydroxycoumarin	3.68	163.0389	C_9_H_6_O_3_	−0.43	1.6	1.3	↑	1.1 × 10^−1^	135.0442, 107.0495, 95.0498, 79.0391	2
coumaranone	3.68	135.0440	C_8_H_6_O_2_	−0.41	1.5	1.3	↑	1.4 × 10^−1^	117.0339, 107.0491, 89.0386	2

* ↑ = up-regulated in RV^HIGH^ ↓ = down-regulated in RV^HIGH.^

**Table 4:** Metabolites identified in UHPLC-HRMS ESI- data that were significant (VIP > 1.5) in the OPLSDA model between RV^HIGH^ compared to RV^LOW^ apple spur buds with associated effect size. *p* values indicate those metabolites that are significant in the linear model with the (y ~ returnbloom) for RV^LOW^, RV^MID^ and RV^HIGH^.

Identity	Retention Time (min)	Mass (m/z) [M − H] −	Molecular Formula	Mass Error (ppm)	VIP Score	Effect Size	*	*p* Value	MS^2^ Ions	Metabolite Level
L-histidine	1.14	154.0613	C_6_H_9_N_3_O_2_	^−^5.84	3.1	2.8	↓	1.7 × 10^−3^	154.0614, 137.0348, 93.0448, 80.0691	2
L-aspartate	1.20	132.0292	C_4_H_7_NO_4_	^−^7.81	2.0	1.3	↓	5.5 × 10^−2^	-	3
aspartyl-histidine	1.21	269.0881	C_10_H_14_N_4_O_5_	^−^3.88	2.7	1.3	↑	2.1 × 10^−3^	-	3
2,5-dihydroxybenzoic acid 2-O-β-D-glucoside	2.96	315.0730	C_13_H_16_O_9_	2.70	1.8	1.4	↑	3.2 × 10^−2^	-	3
dihydro-trans-o-coumaric acid 2-glucoside	3.52	327.1092	C_15_H_20_O_8_	2.02	2.2	1.2	↑	2.6 × 10^−2^	181.0714, 165.0550, 145.0287, 119.0494	2
quinic acid	3.68	191.0556	C_7_H_12_O_6_	^−^2.68	1.5	1.4	↑	1.1 × 10^−1^	191.0556, 173.0449, 127.0391, 82.0284	2
chlorogenic acid	3.68	353.0882	C_16_H_18_O_9_	1.12	1.8	1.4	↑	7.9 × 10^−2^	191.0556, 353.0886, 173.0450	2
phlorisobutanophenone glycoside	3.70	357.1193	C_16_H_22_O_9_	0.54	2.0	1.2	↑	4.5 × 10^−2^	311.0544, 289.0723, 195.0659	2
1-O-feruloylglucose	3.97	355.1041	C_16_H_20_O_9_	1.82	1.6	1.4	↑	9.5 × 10^−2^	177.0546, 147.0443, 137.0598, 119.0495	2

* ↑ = up-regulated in RV^HIGH^ ↓ = down-regulated in RV^HIGH.^

**Table 5:** The molecular formula, accurate mass, [M + H] + and retention time of individual plant hormones and structural derivatives.

Name	Molecular Formula	Acurate Mass	[M + H] +	Retention Time (min)
Methyl (+/−)-jasmonate	C_13_H_20_O_3_	224.1412	224.1412	8.13
Gibberellic acid	C_19_H_22_O_6_	346.1416	347.1495	4.65
Adenine	C_5_H_5_N_5_	135.0545	136.0623	1.32
Adenosine	C_10_H_13_N_5_O_4_	267.0968	268.1046	1.33
Indole-3-acetonitrile	C_10_H_8_N_2_	156.0687	157.0766	6.65
Indole-3-acetic acid	C_10_H_9_NO_2_	175.0633	176.0712	5.68
2-oxindole-3-acetic acid	C_10_H_9_NO_3_	191.0582	192.0661	4.42
Tryptophan	C_11_H_12_N_2_O_2_	204.0899	205.0977	3.51
(+/−)-Abscisic acid	C_15_H_20_O_4_	264.1362	265.1440	5.95
SA	C_7_H_6_O_3_	138.0317	139.0395	5.87
Methyl-indole-3-acetic acid	C_11_H_11_NO_2_	189.0790	190.0868	7.10
Tryptamine	C_10_H_12_N_2_	160.1000	161.1078	3.82

## Data Availability

The datasets generated during the current study are available from the corresponding author upon reasonable request.

## Supplementary Materials

The following supporting information can be downloaded at: https://www.mdpi.com/article/10.3390/ijms23116019/s1.
