# Supplementary material for: Metabolic Pathways for Observed Impacts of Crop Load on Floral Induction in Apple
Source: Int J Mol Sci. 2022 May 27;23(11):6019. doi: 10.3390/ijms23116019 (PMC9181029; doi:10.3390/ijms23116019)
Supplement: Supplementary file 1 [file ijms-23-06019-s001.zip › ijms-1694272-supplementary.pdf]

OFFICIAL

High-throughput analysis of amino acids for protein quantification in plant and animal-derived samples using high resolution mass spectrometry

Priyanka Reddy [1], Tim Plozza [1], Vilnis Ezernieks [1], Dario Stefanelli [2], Alessio Scalisi [3], Ian Goodwin [3,4] and Simone Rochfort [1,5,*]

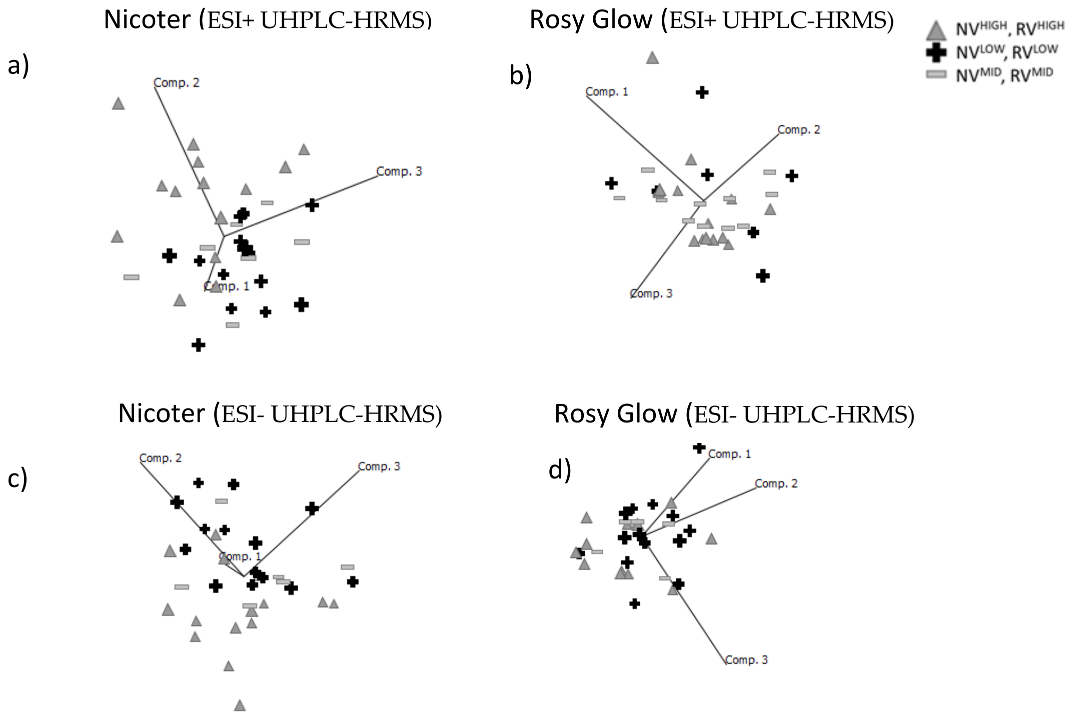

Supplementary Figure S1. PCA scores plot of ESI+ UHPLC-HRMS of (a) Nicoter and (b) Rosy glow and ESI- UHPLC-HRMS of c) Nicoter and b) Rosy Glow, acquired from the aqueous extracts of apple spur buds of 'Nicoter' crop load treatments: NV^{HIGH} (1-2 fruit cm^2 TCSA; *n*=14), NV^{MID} (4.43 fruit cm^2 TCSA; *n*=7) and NV^{LOW} (6-7 fruit cm^2 TCSA; *n*=14) and 'Rosy Glow' crop load treatments: RV^{HIGH} (1-4 fruit cm^2 TCSA; *n*=13), RV^{MID} (8.77 fruit cm^2 TCSA; *n*=6) and RV^{LOW} (11-14 fruit cm^2 TCSA; *n*=14).

OFFICIAL

Supplementary Table S1. P-values associated with the linear model (y (metabolite response) ~ return bloom) for 'Nicoter' (NV^{HIGH}, NV^{MID}, NV^{LOW}) and T-test and fold change of NV^{HIGH} vs NV^{LOW} of the targeted phytohormones and structural derivatives.

Plant hormone	Linear model P-value (Nicoter)	NV^{HIGH} vs NV^{LOW} T-test (***p*** Value)	Fold change of NV^{HIGH} vs NV^{LOW}
adenine	0.460	0.480	0.035
adenosine	0.364	0.367	-0.208
tryptophan	0.054	0.086	-0.310
2-oxindole-3-acetic acid	0.096	0.085	0.303
gibberellic acid	0.650	0.703	0.063
indole-3-acetic acid	0.656	0.849	0.049
salicylic acid	0.416	0.411	0.085
abscisic acid	0.007	0.011	0.266
indole-3-acetonitrile	0.412	0.754	-0.124
methyl-indole-3-acetic acid	0.929	0.497	0.129
methyl jasmonate	0.899	0.857	-0.057
tryptamine	ND	ND	ND

Supplementary Table S2. P-values associated with the linear model (y (metabolite response) ~ return bloom) for 'Rosy Glow' (RV^{HIGH}, RV^{MID}, RV^{LOW}) and T-test and fold change of RV^{HIGH} vs RV^{LOW} of the targeted phytohormones and structural derivatives.

Plant hormone	Linear model P-value (Rosy Glow)	RV^{HIGH} vs RV^{LOW} T-test (***p*** Value)	Fold change of RV^{HIGH} vs RV^{LOW}
adenine	0.188	0.331	0.102
adenosine	0.892	0.849	0.033
tryptophan	0.232	0.197	-0.254
2-oxindole-3-acetic acid	0.504	0.500	0.081
gibberellic acid	0.223	0.212	-0.080
indole-3-acetic acid	0.773	0.874	-0.049
salicylic acid	0.181	0.224	0.189
abscissic acid	0.310	0.546	0.043
indole-3-acetonitrile	0.590	0.530	-0.054
methyl-indole-3-acetic acid	0.546	0.274	0.201
methyl jasmonate	0.517	0.344	-0.142
tryptamine	ND	ND	ND

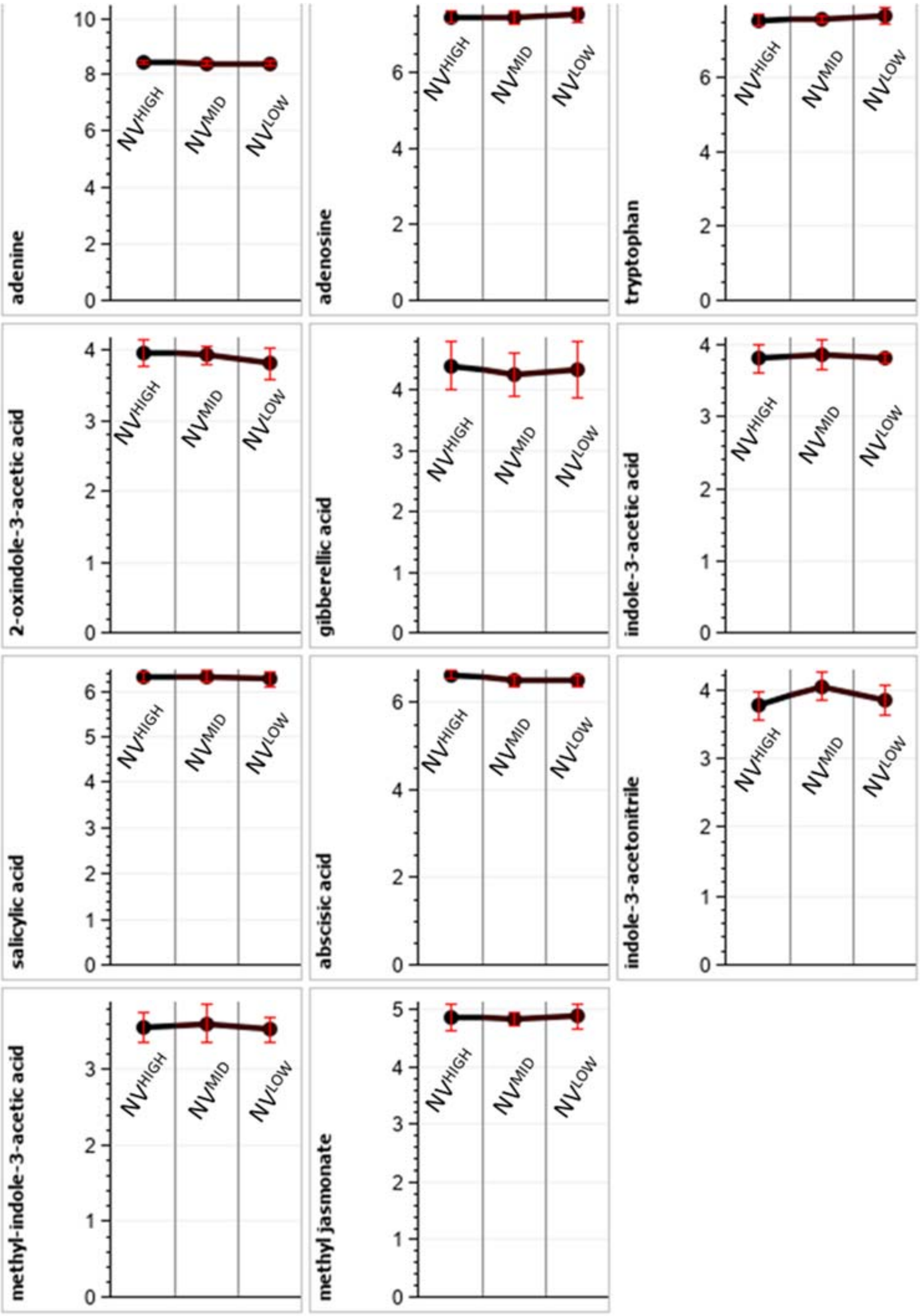

Supplementary Figure S2. Line graphs comparing relative abundances (log10 transformed) of NV^{HIGH}, NV^{MID}, NV^{LOW} treatments of individual target plant hormones and related structural derivatives. Tryptamine was not detected in the samples. All data are mean ±SD.

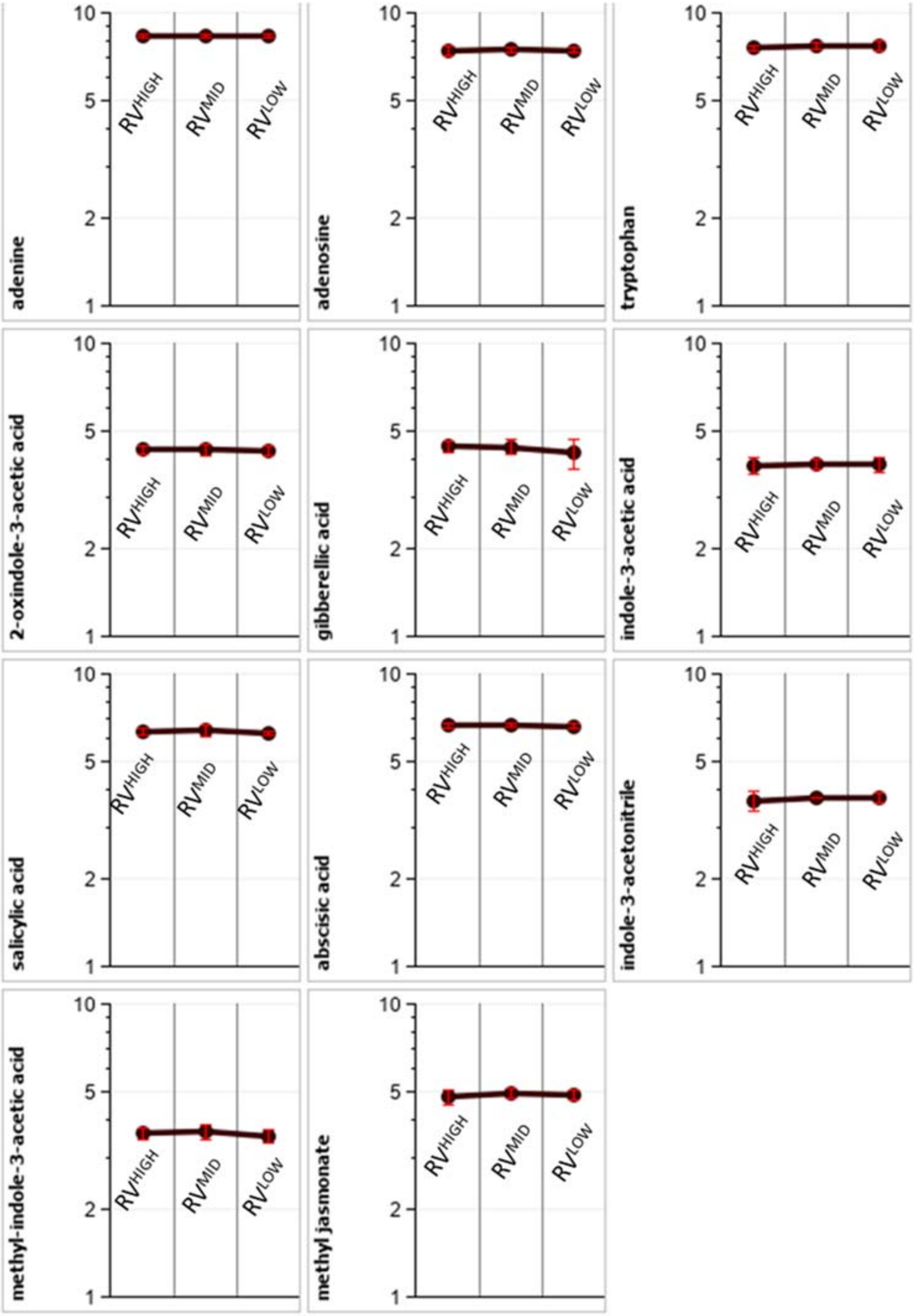

Supplementary Figure S3. Line graphs comparing relative abundances (log10 transformed) of RV^{HIGH}, RV^{MID}, RV^{LOW} treatments of individual target plant hormones and related structural derivatives. Tryptamine was not detected in the samples. All data are mean ±SD.
